# (Cinnamato-κ^2^ *O*,*O*′)(5,5,7,12,12,14-hexa­methyl-1,4,8,11-tetra­aza­cyclo­tetra­decane-κ^4^ *N*,*N*′,*N*′′,*N*′′′)nickel(II) perchlorate monohydrate

**DOI:** 10.1107/S1600536812032175

**Published:** 2012-07-21

**Authors:** Qiang Gao, Yi-Cheng Cao

**Affiliations:** a School of Bioscience and Bioengineering, South China University of Technology, Guangzhou Guangdong 510006, People’s Republic of China

## Abstract

In the title compound, [Ni(C_9_H_7_O_2_)(C_16_H_36_N_4_)]ClO_4_·H_2_O, the macrocyclic 5,5,7,12,12,14-hexa­methyl-1,4,8,11-tetra­aza­cyclo­tetra­decane ligand (*L*) folds around the Ni^II^ atom, which is also chelated by the carboxyl­ate group. The geometry is a distorted N_4_O_2_ octa­hedron. In the crystal, adjacent mol­ecules are connected by O—H⋯O and N—H⋯O hydrogen bonds into a zigzag chain parallel to [010].

## Related literature

For background to this study, see: Tait & Busch (1976 ▶); Curtis (1965 ▶). For related structures, see: Ou *et al.* (2008 ▶, 2009*a* ▶,*b* ▶); Ou & Ng 2010*a* ▶,*b* ▶).
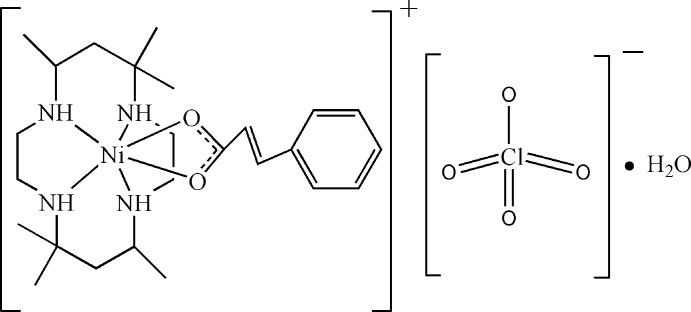


## Experimental

### 

#### Crystal data

[Ni(C_9_H_7_O_2_)(C_16_H_36_N_4_)]ClO_4_·H_2_O
*M* *_r_* = 607.81
Monoclinic, 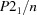

*a* = 10.6903 (11) Å
*b* = 14.5396 (8) Å
*c* = 19.2498 (12) Å
β = 94.225 (6)°
*V* = 2983.9 (4) Å^3^
*Z* = 4
Cu *K*α radiation
μ = 2.16 mm^−1^
*T* = 153 K
0.42 × 0.21 × 0.16 mm

#### Data collection

Agilent Xcalibur Atlas Gemini ultra diffractometer
Absorption correction: multi-scan (*CrysAlis PRO*; Agilent, 2011 ▶) *T*_min_ = 0.464, *T*_max_ = 0.724
10750 measured reflections
5001 independent reflections
4533 reflections with *I* > 2σ(*I*)
*R*_int_ = 0.022

#### Refinement

*R*[*F* ^2^ > 2σ(*F* ^2^)] = 0.038
*wR*(*F* ^2^) = 0.098
*S* = 1.04
5001 reflections
355 parameters
H atoms treated by a mixture of independent and constrained refinement
Δρ_max_ = 0.67 e Å^−3^
Δρ_min_ = −0.53 e Å^−3^

### 

Data collection: *CrysAlis PRO* (Agilent, 2011 ▶); cell refinement: *CrysAlis PRO*; data reduction: *CrysAlis PRO*; program(s) used to solve structure: *SHELXS97* (Sheldrick, 2008 ▶); program(s) used to refine structure: *SHELXL97* (Sheldrick, 2008 ▶); molecular graphics: *SHELXTL* (Sheldrick, 2008 ▶); software used to prepare material for publication: *SHELXTL*.

## Supplementary Material

Crystal structure: contains datablock(s) I, global. DOI: 10.1107/S1600536812032175/ds2203sup1.cif

Structure factors: contains datablock(s) I. DOI: 10.1107/S1600536812032175/ds2203Isup2.hkl

Additional supplementary materials:  crystallographic information; 3D view; checkCIF report

## Figures and Tables

**Table 1:** Hydrogen-bond geometry (Å, °)

*D*—H⋯*A*	*D*—H	H⋯*A*	*D*⋯*A*	*D*—H⋯*A*
N4—H4*D*⋯O1*W* ^i^	0.93	2.11	3.009 (2)	163
N2—H2*C*⋯O1*W* ^i^	0.93	2.19	3.073 (2)	158
O1*W*—H1*WA*⋯O1^ii^	0.80 (3)	1.94 (3)	2.732 (2)	173 (3)
O1*W*—H1*WB*⋯O5	0.80 (3)	2.13 (3)	2.921 (3)	171 (3)

Symmetry codes: (i) 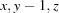; (ii) 
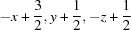
.

**Fig. 1.**
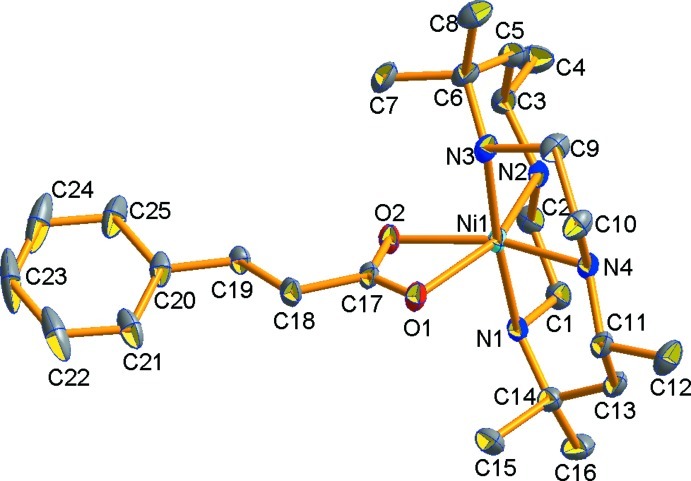
The molecular structure of (I), with atom labels and 30% probability displacement ellipsoids for non-H atoms.

**Fig. 2.**
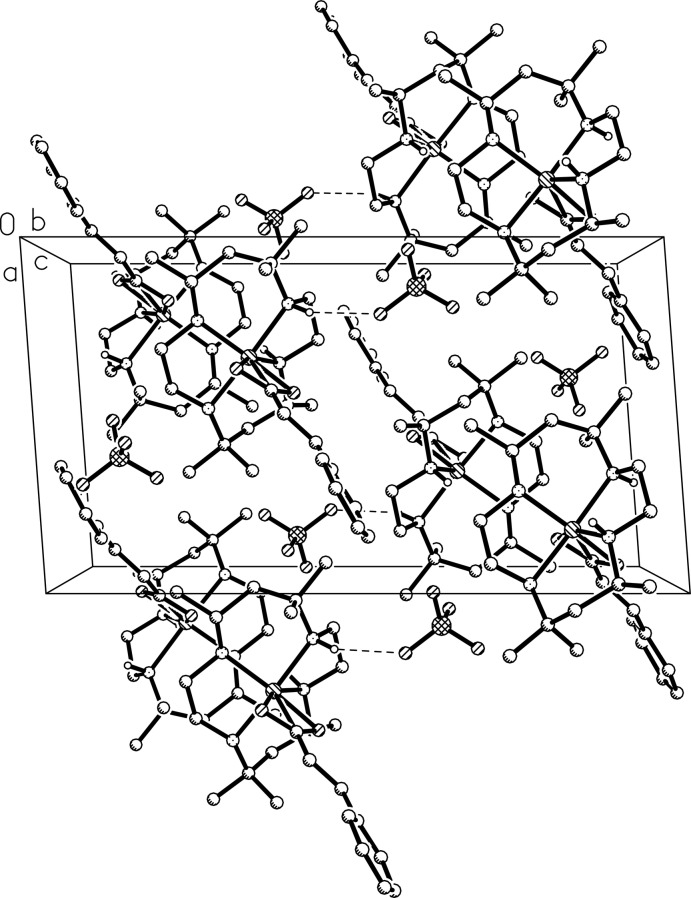
A view of the packing of the title compound

**Fig. 3.**
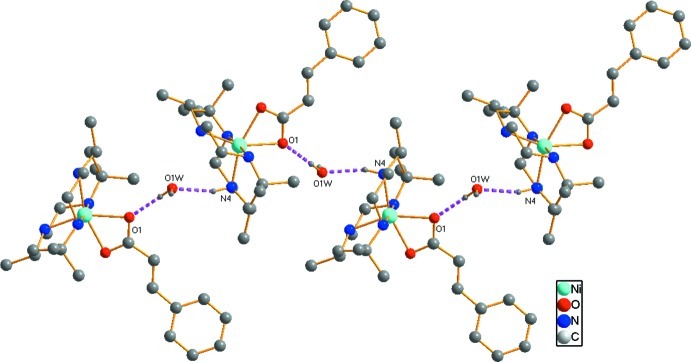
Hydrogen bonding (dashed lines) of the title compound

## supplementary crystallographic information

### Comment

Similar other nickel salts involving the macrocyclic ligand *L* were reported (Ou *et al.*, 2008, 2009*a*, 2009*b*, 2010*a*, 2010*b*). The asymmetric unit in the title compound contains one [NiL(C_6_H_5_C_2_H_2_CO_2_)]^+^, one [ClO_4_]^-^ and one free water molecule. In each cation (Fig. 1), the nickel(II) ion displays a distorted octahedral coordination geometry by coordination with four nitrogen atoms of *L* in a folded conformation, and two carboxylate oxygen atoms of cinnamic acid in *cis* position. Adjacent molecules are connected through the O—H···O (2.732 (2)–2.921 (3) Å) and N—H···O (3.009 (2)–3.073 (2) Å) (Table 1) hydrogen bonding interactions between the carboxylate oxygen atom of cinnamic acid, oxygen atom of water molecule and secondary amine of *L*, generating a zigzag chain (Figs. 2, 3).

### Experimental

A solution of [Ni(rac-*L*)](ClO_4_)_2_ (0.541 g, 1 mmol) in acetonitrile (10 ml) was added to a solution of cinnamic acid (0.148 g, 1 mmol) and NaOH (0.040 g, 1 mmol) in 10 ml of water. The resultant blue solution was evaporated slowly at room temperature. After several weeks, violet prism-shaped crystals were obtained.

### Refinement

H atoms bound to carbon, oxygen and nitrogen atoms were positioned geometrically and refined using the riding model, and with C—H = 0.95 to 1.00 Å, O—H = 0.80 Å and N—H = 0.93 Å, and with *U*(H) set to 1.2 to 1.5 *U*_eq_(C, O, N).

### Crystal data

**Table:**

[Ni(C_9_H_7_O_2_)(C_16_H_36_N_4_)]ClO_4_·H_2_O	*F*(000) = 1296
*M**_r_* = 607.81	*D*_x_ = 1.353 Mg m^−^^3^
Monoclinic, *P*2_1_/*n*	Cu *K*α radiation, λ = 1.54184 Å
Hall symbol: -P 2yn	Cell parameters from 10750 reflections
*a* = 10.6903 (11) Å	θ = 3.8–65.5°
*b* = 14.5396 (8) Å	µ = 2.16 mm^−^^1^
*c* = 19.2498 (12) Å	*T* = 153 K
β = 94.225 (6)°	Prism, violet
*V* = 2983.9 (4) Å^3^	0.42 × 0.21 × 0.16 mm
*Z* = 4	

### Data collection

**Table:**

Agilent Xcalibur Atlas Gemini ultra diffractometer	5001 independent reflections
Radiation source: fine-focus sealed tube	4533 reflections with *I* > 2σ(*I*)
Graphite monochromator	*R*_int_ = 0.022
ω scans	θ_max_ = 65.5°, θ_min_ = 3.8°
Absorption correction: multi-scan (*CrysAlis PRO*; Agilent, 2011)	*h* = −9→12
*T*_min_ = 0.464, *T*_max_ = 0.724	*k* = −15→16
10750 measured reflections	*l* = −20→22

### Refinement

**Table:**

Refinement on *F*^2^	Primary atom site location: structure-invariant direct methods
Least-squares matrix: full	Secondary atom site location: difference Fourier map
*R*[*F*^2^ > 2σ(*F*^2^)] = 0.038	Hydrogen site location: inferred from neighbouring sites
*wR*(*F*^2^) = 0.098	H atoms treated by a mixture of independent and constrained refinement
*S* = 1.04	*w* = 1/[σ^2^(*F*_o_^2^) + (0.0494*P*)^2^ + 2.0261*P*] where *P* = (*F*_o_^2^ + 2*F*_c_^2^)/3
5001 reflections	(Δ/σ)_max_ = 0.001
355 parameters	Δρ_max_ = 0.67 e Å^−^^3^
0 restraints	Δρ_min_ = −0.53 e Å^−^^3^

### Special details

**Table:**

Experimental. Absorption correction: CrysAlisPro, Agilent Technologies, Version 1.171.35.15 Empirical absorption correction using spherical harmonics, implemented in SCALE3 ABSPACK scaling algorithm.
Geometry. All e.s.d.'s (except the e.s.d. in the dihedral angle between two l.s. planes) are estimated using the full covariance matrix. The cell e.s.d.'s are taken into account individually in the estimation of e.s.d.'s in distances, angles and torsion angles; correlations between e.s.d.'s in cell parameters are only used when they are defined by crystal symmetry. An approximate (isotropic) treatment of cell e.s.d.'s is used for estimating e.s.d.'s involving l.s. planes.
Refinement. Refinement of *F*^2^ against ALL reflections. The weighted *R*-factor *wR* and goodness of fit *S* are based on *F*^2^, conventional *R*-factors *R* are based on *F*, with *F* set to zero for negative *F*^2^. The threshold expression of *F*^2^ > σ(*F*^2^) is used only for calculating *R*-factors(gt) *etc*. and is not relevant to the choice of reflections for refinement. *R*-factors based on *F*^2^ are statistically about twice as large as those based on *F*, and *R*- factors based on ALL data will be even larger.

### Fractional atomic coordinates and isotropic or equivalent isotropic displacement parameters (Å^2^)

**Table:**

	*x*	*y*	*z*	*U*_iso_*/*U*_eq_	
Ni1	0.82693 (3)	0.10829 (2)	0.168866 (17)	0.01820 (11)	
Cl1	0.37923 (5)	0.79143 (4)	0.11605 (3)	0.03427 (15)	
O1W	0.65932 (16)	0.88699 (11)	0.21653 (9)	0.0329 (4)	
N4	0.71496 (15)	0.08543 (12)	0.25076 (9)	0.0215 (4)	
H4D	0.6875	0.0248	0.2477	0.026*	
N3	0.98016 (15)	0.07735 (12)	0.24375 (9)	0.0226 (4)	
H3A	1.0142	0.1338	0.2578	0.027*	
N1	0.66694 (16)	0.12951 (11)	0.09671 (9)	0.0224 (4)	
H1C	0.6945	0.1679	0.0623	0.027*	
O1	0.86371 (13)	0.25222 (10)	0.18630 (7)	0.0246 (3)	
O2	0.93361 (13)	0.17771 (9)	0.09782 (7)	0.0234 (3)	
N2	0.83993 (15)	−0.01980 (11)	0.12133 (8)	0.0199 (4)	
H2C	0.7974	−0.0624	0.1469	0.024*	
C7	1.1671 (2)	0.07934 (17)	0.17803 (13)	0.0336 (5)	
H7A	1.1139	0.1024	0.1382	0.050*	
H7B	1.2356	0.0426	0.1612	0.050*	
H7C	1.2021	0.1313	0.2054	0.050*	
C18	0.99490 (19)	0.33452 (14)	0.11318 (11)	0.0254 (5)	
H18	0.9782	0.3914	0.1349	0.030*	
C9	0.9196 (2)	0.04063 (18)	0.30456 (11)	0.0305 (5)	
H9A	0.9776	0.0462	0.3469	0.037*	
H9B	0.8998	−0.0253	0.2972	0.037*	
C13	0.51412 (19)	0.13204 (15)	0.18830 (12)	0.0271 (5)	
H13A	0.5027	0.0651	0.1809	0.033*	
H13B	0.4314	0.1574	0.1982	0.033*	
O5	0.4206 (2)	0.88266 (13)	0.13241 (12)	0.0614 (6)	
C12	0.5291 (2)	0.12492 (19)	0.31703 (13)	0.0379 (6)	
H12A	0.4976	0.0616	0.3144	0.057*	
H12B	0.4584	0.1677	0.3178	0.057*	
H12C	0.5844	0.1326	0.3596	0.057*	
O3	0.29831 (17)	0.79250 (13)	0.05395 (9)	0.0459 (5)	
O6	0.3166 (3)	0.75420 (18)	0.17151 (11)	0.0832 (8)	
C6	1.08899 (19)	0.01963 (15)	0.22362 (11)	0.0266 (5)	
C11	0.60231 (19)	0.14496 (15)	0.25364 (11)	0.0258 (5)	
H11	0.6302	0.2106	0.2559	0.031*	
C8	1.1740 (2)	−0.00999 (18)	0.28747 (13)	0.0366 (6)	
H8A	1.1962	0.0440	0.3163	0.055*	
H8B	1.2505	−0.0382	0.2721	0.055*	
H8C	1.1295	−0.0546	0.3148	0.055*	
C5	1.0409 (2)	−0.06705 (15)	0.18454 (11)	0.0265 (5)	
H5A	1.1141	−0.1071	0.1781	0.032*	
H5B	0.9860	−0.1006	0.2151	0.032*	
C14	0.54944 (19)	0.17362 (15)	0.11962 (12)	0.0280 (5)	
C15	0.5743 (2)	0.27698 (16)	0.12686 (14)	0.0378 (6)	
H15A	0.6482	0.2872	0.1592	0.057*	
H15B	0.5013	0.3071	0.1448	0.057*	
H15C	0.5895	0.3028	0.0812	0.057*	
C2	0.7687 (2)	−0.00750 (15)	0.05307 (11)	0.0255 (5)	
H2A	0.7540	−0.0681	0.0306	0.031*	
H2B	0.8180	0.0306	0.0224	0.031*	
C17	0.92733 (18)	0.25108 (14)	0.13254 (11)	0.0224 (4)	
O4	0.4837 (2)	0.73377 (18)	0.10554 (16)	0.0933 (9)	
C16	0.4378 (2)	0.15948 (18)	0.06591 (13)	0.0381 (6)	
H16A	0.4606	0.1801	0.0201	0.057*	
H16B	0.3659	0.1952	0.0797	0.057*	
H16C	0.4157	0.0941	0.0637	0.057*	
C23	1.3114 (4)	0.5501 (3)	0.00822 (16)	0.0737 (12)	
H23	1.3664	0.5986	−0.0024	0.088*	
C1	0.64498 (19)	0.03845 (14)	0.06257 (11)	0.0254 (5)	
H1A	0.5981	0.0468	0.0167	0.031*	
H1B	0.5941	−0.0008	0.0917	0.031*	
C20	1.1556 (2)	0.40741 (16)	0.04353 (11)	0.0275 (5)	
C24	1.3420 (3)	0.4605 (3)	−0.00655 (14)	0.0672 (11)	
H24	1.4168	0.4479	−0.0286	0.081*	
C21	1.1246 (3)	0.49896 (16)	0.05564 (12)	0.0368 (6)	
H21	1.0489	0.5128	0.0763	0.044*	
C10	0.8008 (2)	0.09348 (17)	0.31431 (11)	0.0307 (5)	
H10A	0.7598	0.0687	0.3548	0.037*	
H10B	0.8210	0.1590	0.3235	0.037*	
C25	1.2639 (2)	0.3878 (2)	0.01061 (12)	0.0411 (6)	
H25	1.2847	0.3261	−0.0001	0.049*	
C4	0.9680 (2)	−0.14652 (17)	0.07386 (13)	0.0389 (6)	
H4A	0.9174	−0.1917	0.0971	0.058*	
H4B	1.0540	−0.1695	0.0727	0.058*	
H4C	0.9319	−0.1367	0.0262	0.058*	
C3	0.96907 (19)	−0.05558 (14)	0.11380 (11)	0.0243 (4)	
H3	1.0147	−0.0093	0.0868	0.029*	
C22	1.2023 (3)	0.5696 (2)	0.03801 (14)	0.0595 (10)	
H22	1.1800	0.6316	0.0465	0.071*	
C19	1.07876 (19)	0.33104 (14)	0.06586 (11)	0.0228 (4)	
H19	1.0902	0.2732	0.0443	0.027*	
H1WA	0.657 (2)	0.8500 (19)	0.2470 (14)	0.034*	
H1WB	0.597 (3)	0.8806 (18)	0.1911 (14)	0.034*	

### Atomic displacement parameters (Å^2^)

**Table:**

	*U*^11^	*U*^22^	*U*^33^	*U*^12^	*U*^13^	*U*^23^
Ni1	0.01681 (19)	0.01662 (19)	0.02133 (19)	−0.00561 (13)	0.00246 (13)	−0.00172 (13)
Cl1	0.0359 (3)	0.0264 (3)	0.0385 (3)	−0.0035 (2)	−0.0111 (2)	0.0074 (2)
O1W	0.0335 (9)	0.0305 (9)	0.0336 (9)	−0.0151 (7)	−0.0042 (7)	0.0111 (7)
N4	0.0191 (8)	0.0208 (9)	0.0248 (9)	−0.0045 (7)	0.0038 (7)	−0.0013 (7)
N3	0.0202 (9)	0.0248 (9)	0.0229 (9)	−0.0049 (7)	0.0026 (7)	−0.0053 (7)
N1	0.0225 (9)	0.0182 (9)	0.0263 (9)	−0.0052 (7)	0.0008 (7)	0.0026 (7)
O1	0.0242 (7)	0.0212 (7)	0.0293 (8)	−0.0070 (6)	0.0086 (6)	−0.0051 (6)
O2	0.0259 (7)	0.0185 (7)	0.0263 (7)	−0.0071 (6)	0.0058 (6)	−0.0049 (6)
N2	0.0193 (8)	0.0197 (8)	0.0205 (8)	−0.0054 (7)	0.0008 (7)	−0.0008 (7)
C7	0.0186 (11)	0.0387 (13)	0.0441 (14)	−0.0068 (10)	0.0061 (10)	−0.0040 (11)
C18	0.0269 (11)	0.0164 (10)	0.0334 (12)	−0.0068 (9)	0.0058 (9)	−0.0038 (9)
C9	0.0253 (11)	0.0448 (14)	0.0211 (11)	0.0014 (10)	0.0008 (9)	0.0028 (10)
C13	0.0173 (10)	0.0253 (11)	0.0391 (13)	−0.0035 (8)	0.0038 (9)	−0.0034 (10)
O5	0.0721 (14)	0.0309 (10)	0.0761 (15)	−0.0172 (10)	−0.0277 (12)	0.0059 (10)
C12	0.0270 (12)	0.0488 (15)	0.0394 (14)	−0.0043 (11)	0.0138 (10)	−0.0077 (12)
O3	0.0502 (11)	0.0433 (11)	0.0412 (10)	−0.0115 (9)	−0.0168 (8)	0.0114 (8)
O6	0.121 (2)	0.0837 (18)	0.0447 (13)	−0.0476 (16)	0.0012 (13)	0.0221 (12)
C6	0.0177 (10)	0.0306 (12)	0.0314 (12)	−0.0001 (9)	0.0008 (9)	−0.0048 (9)
C11	0.0203 (10)	0.0228 (11)	0.0351 (12)	−0.0038 (9)	0.0067 (9)	−0.0027 (9)
C8	0.0232 (11)	0.0477 (15)	0.0376 (13)	0.0025 (10)	−0.0056 (10)	−0.0055 (11)
C5	0.0218 (10)	0.0264 (11)	0.0311 (12)	0.0022 (9)	0.0006 (9)	−0.0022 (9)
C14	0.0208 (10)	0.0236 (11)	0.0391 (13)	0.0001 (9)	−0.0009 (9)	0.0024 (10)
C15	0.0353 (13)	0.0215 (12)	0.0563 (16)	0.0028 (10)	0.0016 (11)	0.0055 (11)
C2	0.0278 (11)	0.0241 (11)	0.0235 (11)	−0.0029 (9)	−0.0045 (8)	−0.0040 (9)
C17	0.0189 (10)	0.0195 (10)	0.0289 (11)	−0.0066 (8)	0.0031 (8)	−0.0013 (9)
O4	0.0641 (15)	0.0748 (17)	0.136 (3)	0.0368 (14)	−0.0290 (16)	−0.0102 (16)
C16	0.0265 (12)	0.0396 (14)	0.0468 (15)	0.0053 (11)	−0.0062 (10)	0.0038 (11)
C23	0.097 (3)	0.085 (3)	0.0373 (16)	−0.071 (2)	−0.0065 (17)	0.0221 (17)
C1	0.0245 (11)	0.0227 (11)	0.0278 (11)	−0.0059 (9)	−0.0065 (9)	−0.0022 (9)
C20	0.0298 (12)	0.0336 (12)	0.0185 (10)	−0.0150 (10)	−0.0024 (8)	0.0028 (9)
C24	0.0511 (18)	0.121 (3)	0.0305 (14)	−0.052 (2)	0.0080 (12)	0.0054 (17)
C21	0.0532 (15)	0.0282 (12)	0.0274 (12)	−0.0194 (11)	−0.0085 (11)	0.0051 (10)
C10	0.0257 (11)	0.0440 (14)	0.0229 (11)	0.0004 (10)	0.0041 (9)	−0.0024 (10)
C25	0.0330 (13)	0.0656 (18)	0.0248 (12)	−0.0198 (12)	0.0034 (10)	0.0052 (12)
C4	0.0389 (13)	0.0328 (13)	0.0437 (14)	0.0087 (11)	−0.0050 (11)	−0.0144 (11)
C3	0.0220 (10)	0.0238 (11)	0.0273 (11)	−0.0012 (9)	0.0030 (8)	−0.0042 (9)
C22	0.097 (3)	0.0437 (17)	0.0341 (14)	−0.0435 (17)	−0.0206 (16)	0.0173 (13)
C19	0.0238 (10)	0.0198 (10)	0.0247 (10)	−0.0067 (8)	0.0004 (8)	−0.0022 (8)

### Geometric parameters (Å, º)

**Table:**

Ni1—N4	2.0752 (17)	C12—H12C	0.9800
Ni1—N2	2.0841 (17)	C6—C8	1.535 (3)
Ni1—O2	2.1023 (14)	C6—C5	1.537 (3)
Ni1—N1	2.1447 (17)	C11—H11	1.0000
Ni1—N3	2.1485 (17)	C8—H8A	0.9800
Ni1—O1	2.1512 (14)	C8—H8B	0.9800
Ni1—C17	2.462 (2)	C8—H8C	0.9800
Cl1—O6	1.409 (2)	C5—C3	1.522 (3)
Cl1—O4	1.422 (2)	C5—H5A	0.9900
Cl1—O3	1.4231 (17)	C5—H5B	0.9900
Cl1—O5	1.4260 (19)	C14—C15	1.531 (3)
O1W—H1WA	0.80 (3)	C14—C16	1.534 (3)
O1W—H1WB	0.80 (3)	C15—H15A	0.9800
N4—C10	1.479 (3)	C15—H15B	0.9800
N4—C11	1.487 (3)	C15—H15C	0.9800
N4—H4D	0.9300	C2—C1	1.505 (3)
N3—C9	1.478 (3)	C2—H2A	0.9900
N3—C6	1.508 (3)	C2—H2B	0.9900
N3—H3A	0.9300	C16—H16A	0.9800
N1—C1	1.489 (3)	C16—H16B	0.9800
N1—C14	1.505 (3)	C16—H16C	0.9800
N1—H1C	0.9300	C23—C22	1.367 (5)
O1—C17	1.280 (2)	C23—C24	1.378 (6)
O2—C17	1.263 (2)	C23—H23	0.9500
N2—C2	1.480 (3)	C1—H1A	0.9900
N2—C3	1.492 (3)	C1—H1B	0.9900
N2—H2C	0.9300	C20—C25	1.390 (3)
C7—C6	1.526 (3)	C20—C21	1.395 (3)
C7—H7A	0.9800	C20—C19	1.464 (3)
C7—H7B	0.9800	C24—C25	1.402 (4)
C7—H7C	0.9800	C24—H24	0.9500
C18—C19	1.325 (3)	C21—C22	1.379 (4)
C18—C17	1.474 (3)	C21—H21	0.9500
C18—H18	0.9500	C10—H10A	0.9900
C9—C10	1.508 (3)	C10—H10B	0.9900
C9—H9A	0.9900	C25—H25	0.9500
C9—H9B	0.9900	C4—C3	1.529 (3)
C13—C14	1.526 (3)	C4—H4A	0.9800
C13—C11	1.527 (3)	C4—H4B	0.9800
C13—H13A	0.9900	C4—H4C	0.9800
C13—H13B	0.9900	C3—H3	1.0000
C12—C11	1.525 (3)	C22—H22	0.9500
C12—H12A	0.9800	C19—H19	0.9500
C12—H12B	0.9800		
			
N4—Ni1—N2	104.55 (7)	N4—C11—H11	108.5
N4—Ni1—O2	160.39 (6)	C12—C11—H11	108.5
N2—Ni1—O2	94.96 (6)	C13—C11—H11	108.5
N4—Ni1—N1	92.18 (7)	C6—C8—H8A	109.5
N2—Ni1—N1	85.41 (6)	C6—C8—H8B	109.5
O2—Ni1—N1	87.40 (6)	H8A—C8—H8B	109.5
N4—Ni1—N3	84.88 (6)	C6—C8—H8C	109.5
N2—Ni1—N3	92.02 (7)	H8A—C8—H8C	109.5
O2—Ni1—N3	96.52 (6)	H8B—C8—H8C	109.5
N1—Ni1—N3	175.49 (6)	C3—C5—C6	118.46 (18)
N4—Ni1—O1	98.41 (6)	C3—C5—H5A	107.7
N2—Ni1—O1	157.00 (6)	C6—C5—H5A	107.7
O2—Ni1—O1	62.14 (5)	C3—C5—H5B	107.7
N1—Ni1—O1	95.11 (6)	C6—C5—H5B	107.7
N3—Ni1—O1	88.70 (6)	H5A—C5—H5B	107.1
N4—Ni1—C17	129.70 (7)	N1—C14—C13	110.50 (17)
N2—Ni1—C17	125.75 (7)	N1—C14—C15	107.56 (17)
O2—Ni1—C17	30.87 (6)	C13—C14—C15	111.3 (2)
N1—Ni1—C17	92.19 (7)	N1—C14—C16	111.70 (18)
N3—Ni1—C17	92.31 (7)	C13—C14—C16	107.52 (18)
O1—Ni1—C17	31.29 (6)	C15—C14—C16	108.32 (19)
O6—Cl1—O4	107.63 (19)	C14—C15—H15A	109.5
O6—Cl1—O3	110.32 (13)	C14—C15—H15B	109.5
O4—Cl1—O3	108.77 (15)	H15A—C15—H15B	109.5
O6—Cl1—O5	110.30 (15)	C14—C15—H15C	109.5
O4—Cl1—O5	110.23 (15)	H15A—C15—H15C	109.5
O3—Cl1—O5	109.56 (11)	H15B—C15—H15C	109.5
H1WA—O1W—H1WB	107 (3)	N2—C2—C1	110.18 (17)
C10—N4—C11	112.21 (16)	N2—C2—H2A	109.6
C10—N4—Ni1	104.95 (12)	C1—C2—H2A	109.6
C11—N4—Ni1	116.68 (13)	N2—C2—H2B	109.6
C10—N4—H4D	107.5	C1—C2—H2B	109.6
C11—N4—H4D	107.5	H2A—C2—H2B	108.1
Ni1—N4—H4D	107.5	O2—C17—O1	119.40 (18)
C9—N3—C6	113.18 (17)	O2—C17—C18	121.02 (18)
C9—N3—Ni1	104.49 (12)	O1—C17—C18	119.56 (18)
C6—N3—Ni1	120.57 (12)	O2—C17—Ni1	58.63 (10)
C9—N3—H3A	105.8	O1—C17—Ni1	60.82 (10)
C6—N3—H3A	105.8	C18—C17—Ni1	176.35 (15)
Ni1—N3—H3A	105.8	C14—C16—H16A	109.5
C1—N1—C14	113.60 (16)	C14—C16—H16B	109.5
C1—N1—Ni1	104.49 (12)	H16A—C16—H16B	109.5
C14—N1—Ni1	120.96 (13)	C14—C16—H16C	109.5
C1—N1—H1C	105.5	H16A—C16—H16C	109.5
C14—N1—H1C	105.5	H16B—C16—H16C	109.5
Ni1—N1—H1C	105.5	C22—C23—C24	120.4 (3)
C17—O1—Ni1	87.89 (11)	C22—C23—H23	119.8
C17—O2—Ni1	90.50 (12)	C24—C23—H23	119.8
C2—N2—C3	112.02 (16)	N1—C1—C2	109.69 (16)
C2—N2—Ni1	103.61 (12)	N1—C1—H1A	109.7
C3—N2—Ni1	116.50 (12)	C2—C1—H1A	109.7
C2—N2—H2C	108.1	N1—C1—H1B	109.7
C3—N2—H2C	108.1	C2—C1—H1B	109.7
Ni1—N2—H2C	108.1	H1A—C1—H1B	108.2
C6—C7—H7A	109.5	C25—C20—C21	119.2 (2)
C6—C7—H7B	109.5	C25—C20—C19	118.8 (2)
H7A—C7—H7B	109.5	C21—C20—C19	122.0 (2)
C6—C7—H7C	109.5	C23—C24—C25	120.7 (3)
H7A—C7—H7C	109.5	C23—C24—H24	119.7
H7B—C7—H7C	109.5	C25—C24—H24	119.7
C19—C18—C17	120.75 (19)	C22—C21—C20	121.0 (3)
C19—C18—H18	119.6	C22—C21—H21	119.5
C17—C18—H18	119.6	C20—C21—H21	119.5
N3—C9—C10	109.66 (18)	N4—C10—C9	109.51 (17)
N3—C9—H9A	109.7	N4—C10—H10A	109.8
C10—C9—H9A	109.7	C9—C10—H10A	109.8
N3—C9—H9B	109.7	N4—C10—H10B	109.8
C10—C9—H9B	109.7	C9—C10—H10B	109.8
H9A—C9—H9B	108.2	H10A—C10—H10B	108.2
C14—C13—C11	119.19 (17)	C20—C25—C24	118.9 (3)
C14—C13—H13A	107.5	C20—C25—H25	120.6
C11—C13—H13A	107.5	C24—C25—H25	120.6
C14—C13—H13B	107.5	C3—C4—H4A	109.5
C11—C13—H13B	107.5	C3—C4—H4B	109.5
H13A—C13—H13B	107.0	H4A—C4—H4B	109.5
C11—C12—H12A	109.5	C3—C4—H4C	109.5
C11—C12—H12B	109.5	H4A—C4—H4C	109.5
H12A—C12—H12B	109.5	H4B—C4—H4C	109.5
C11—C12—H12C	109.5	N2—C3—C5	111.13 (16)
H12A—C12—H12C	109.5	N2—C3—C4	112.18 (17)
H12B—C12—H12C	109.5	C5—C3—C4	109.85 (18)
N3—C6—C7	107.25 (18)	N2—C3—H3	107.8
N3—C6—C8	111.87 (17)	C5—C3—H3	107.8
C7—C6—C8	107.64 (18)	C4—C3—H3	107.8
N3—C6—C5	110.21 (16)	C23—C22—C21	119.8 (3)
C7—C6—C5	111.26 (18)	C23—C22—H22	120.1
C8—C6—C5	108.59 (19)	C21—C22—H22	120.1
N4—C11—C12	112.52 (19)	C18—C19—C20	126.5 (2)
N4—C11—C13	110.49 (17)	C18—C19—H19	116.7
C12—C11—C13	108.28 (17)	C20—C19—H19	116.7
			
N2—Ni1—N4—C10	109.46 (13)	C10—N4—C11—C12	−56.9 (2)
O2—Ni1—N4—C10	−76.4 (2)	Ni1—N4—C11—C12	−177.99 (14)
N1—Ni1—N4—C10	−164.72 (14)	C10—N4—C11—C13	−178.01 (17)
N3—Ni1—N4—C10	18.70 (14)	Ni1—N4—C11—C13	60.86 (19)
O1—Ni1—N4—C10	−69.22 (14)	C14—C13—C11—N4	−74.0 (2)
C17—Ni1—N4—C10	−70.05 (16)	C14—C13—C11—C12	162.3 (2)
N2—Ni1—N4—C11	−125.64 (14)	N3—C6—C5—C3	65.4 (2)
O2—Ni1—N4—C11	48.5 (3)	C7—C6—C5—C3	−53.4 (2)
N1—Ni1—N4—C11	−39.82 (14)	C8—C6—C5—C3	−171.74 (18)
N3—Ni1—N4—C11	143.60 (14)	C1—N1—C14—C13	79.9 (2)
O1—Ni1—N4—C11	55.67 (14)	Ni1—N1—C14—C13	−45.6 (2)
C17—Ni1—N4—C11	54.85 (16)	C1—N1—C14—C15	−158.53 (18)
N4—Ni1—N3—C9	10.68 (14)	Ni1—N1—C14—C15	76.0 (2)
N2—Ni1—N3—C9	−93.76 (14)	C1—N1—C14—C16	−39.8 (2)
O2—Ni1—N3—C9	171.01 (13)	Ni1—N1—C14—C16	−165.28 (15)
N1—Ni1—N3—C9	−38.6 (9)	C11—C13—C14—N1	64.3 (2)
O1—Ni1—N3—C9	109.24 (14)	C11—C13—C14—C15	−55.1 (3)
C17—Ni1—N3—C9	140.33 (14)	C11—C13—C14—C16	−173.57 (19)
N4—Ni1—N3—C6	139.34 (15)	C3—N2—C2—C1	−173.67 (16)
N2—Ni1—N3—C6	34.91 (15)	Ni1—N2—C2—C1	−47.29 (18)
O2—Ni1—N3—C6	−60.32 (15)	Ni1—O2—C17—O1	−2.56 (19)
N1—Ni1—N3—C6	90.1 (8)	Ni1—O2—C17—C18	175.75 (18)
O1—Ni1—N3—C6	−122.09 (15)	Ni1—O1—C17—O2	2.50 (19)
C17—Ni1—N3—C6	−91.00 (15)	Ni1—O1—C17—C18	−175.83 (18)
N4—Ni1—N1—C1	−96.07 (13)	C19—C18—C17—O2	−10.4 (3)
N2—Ni1—N1—C1	8.36 (13)	C19—C18—C17—O1	167.9 (2)
O2—Ni1—N1—C1	103.55 (13)	C19—C18—C17—Ni1	73 (3)
N3—Ni1—N1—C1	−47.0 (9)	N4—Ni1—C17—O2	−175.87 (11)
O1—Ni1—N1—C1	165.28 (12)	N2—Ni1—C17—O2	4.71 (15)
C17—Ni1—N1—C1	134.05 (13)	N1—Ni1—C17—O2	−81.22 (12)
N4—Ni1—N1—C14	33.51 (15)	N3—Ni1—C17—O2	98.87 (12)
N2—Ni1—N1—C14	137.94 (15)	O1—Ni1—C17—O2	−177.45 (19)
O2—Ni1—N1—C14	−126.86 (14)	N4—Ni1—C17—O1	1.57 (15)
N3—Ni1—N1—C14	82.6 (8)	N2—Ni1—C17—O1	−177.85 (10)
O1—Ni1—N1—C14	−65.14 (14)	O2—Ni1—C17—O1	177.45 (19)
C17—Ni1—N1—C14	−96.36 (15)	N1—Ni1—C17—O1	96.23 (12)
N4—Ni1—O1—C17	−178.78 (12)	N3—Ni1—C17—O1	−83.69 (12)
N2—Ni1—O1—C17	4.5 (2)	N4—Ni1—C17—C18	99 (2)
O2—Ni1—O1—C17	−1.48 (11)	N2—Ni1—C17—C18	−81 (2)
N1—Ni1—O1—C17	−85.81 (12)	O2—Ni1—C17—C18	−86 (2)
N3—Ni1—O1—C17	96.60 (12)	N1—Ni1—C17—C18	−167 (2)
N4—Ni1—O2—C17	9.5 (2)	N3—Ni1—C17—C18	13 (2)
N2—Ni1—O2—C17	−176.17 (12)	O1—Ni1—C17—C18	97 (2)
N1—Ni1—O2—C17	98.67 (12)	C14—N1—C1—C2	−170.05 (17)
N3—Ni1—O2—C17	−83.56 (12)	Ni1—N1—C1—C2	−36.21 (18)
O1—Ni1—O2—C17	1.50 (11)	N2—C2—C1—N1	59.0 (2)
N4—Ni1—N2—C2	111.64 (12)	C22—C23—C24—C25	−2.0 (5)
O2—Ni1—N2—C2	−66.40 (12)	C25—C20—C21—C22	−2.6 (3)
N1—Ni1—N2—C2	20.58 (12)	C19—C20—C21—C22	176.0 (2)
N3—Ni1—N2—C2	−163.13 (12)	C11—N4—C10—C9	−173.26 (18)
O1—Ni1—N2—C2	−71.7 (2)	Ni1—N4—C10—C9	−45.6 (2)
C17—Ni1—N2—C2	−68.82 (14)	N3—C9—C10—N4	59.0 (2)
N4—Ni1—N2—C3	−124.88 (14)	C21—C20—C25—C24	2.9 (3)
O2—Ni1—N2—C3	57.08 (14)	C19—C20—C25—C24	−175.7 (2)
N1—Ni1—N2—C3	144.06 (14)	C23—C24—C25—C20	−0.7 (4)
N3—Ni1—N2—C3	−39.65 (14)	C2—N2—C3—C5	179.60 (17)
O1—Ni1—N2—C3	51.8 (2)	Ni1—N2—C3—C5	60.6 (2)
C17—Ni1—N2—C3	54.66 (16)	C2—N2—C3—C4	−57.0 (2)
C6—N3—C9—C10	−171.36 (17)	Ni1—N2—C3—C4	−176.01 (15)
Ni1—N3—C9—C10	−38.4 (2)	C6—C5—C3—N2	−74.0 (2)
C9—N3—C6—C7	−161.79 (17)	C6—C5—C3—C4	161.30 (19)
Ni1—N3—C6—C7	73.53 (19)	C24—C23—C22—C21	2.3 (4)
C9—N3—C6—C8	−44.0 (2)	C20—C21—C22—C23	0.0 (4)
Ni1—N3—C6—C8	−168.65 (15)	C17—C18—C19—C20	−177.7 (2)
C9—N3—C6—C5	77.0 (2)	C25—C20—C19—C18	160.3 (2)
Ni1—N3—C6—C5	−47.7 (2)	C21—C20—C19—C18	−18.4 (3)

### Hydrogen-bond geometry (Å, º)

**Table:**

*D*—H···*A*	*D*—H	H···*A*	*D*···*A*	*D*—H···*A*
N4—H4*D*···O1*W*^i^	0.93	2.11	3.009 (2)	163
N2—H2*C*···O1*W*^i^	0.93	2.19	3.073 (2)	158
O1*W*—H1*WA*···O1^ii^	0.80 (3)	1.94 (3)	2.732 (2)	173 (3)
O1*W*—H1*WB*···O5	0.80 (3)	2.13 (3)	2.921 (3)	171 (3)

Symmetry codes: (i) *x*, *y*−1, *z*; (ii) −*x*+3/2, *y*+1/2, −*z*+1/2.
